# New Paradigms for Virus Detection, Surveillance and Control of Zika Virus Vectors in the Settings of Southeast Asia

**DOI:** 10.3389/fmicb.2016.01452

**Published:** 2016-09-13

**Authors:** Indra Vythilingam, Jamal I-C. Sam, Yoke F. Chan, Loke T. Khaw, Wan Y. Wan Sulaiman

**Affiliations:** ^1^Department of Parasitology, Faculty of Medicine, University of MalayaKuala Lumpur, Malaysia; ^2^Department of Microbiology, Faculty of Medicine, University of MalayaKuala Lumpur, Malaysia

**Keywords:** Zika virus, vectors, diagnostic tools, new paradigms, control

## Abstract

Zika virus (ZIKV) has now become a global public health concern. The vectors for ZIKV are *Aedes aegypti* and *A. albopictus*. Both these mosquitoes are predominant in Southeast Asia and are also responsible for the spread of other arboviral diseases like dengue virus and chikungunya virus. The incidence of dengue has been increasing over the years and this is of concern to public health workers. Simple laboratory tools for the detection of ZIKV is also lacking. In the absence of drugs and vaccine for these arboviral diseases, vector control is the main option for surveillance and control. *Aedes* larval surveys have been the hallmark of dengue control along with larviciding and fogging when cases are reported. However, we need new paradigms and options for control of these vectors. The current situation in Southeast Asia clearly proves that effective strategies for vector control need to be proactive and not reactive. This will be the way forward to control epidemics of these diseases inclusive of ZIKV until a vaccine becomes available.

## Introduction

Zika virus (ZIKV) which was first discovered from the Rhesus monkey in the Zika forest of Uganda in 1947 (Dick et al., 1952) has now become a global public health concern (Fauci and Morens, 2016; Focosi et al., 2016). ZIKV is a flavivirus and is maintained in a sylvatic cycle which involves non-human primates and the *Aedes* mosquitoes as vectors (*Aedes africanus, A. aegypti*) (Haddow et al., 1964; Marchette et al., 1969). ZIKV was only known to cause infection in Africa and Southeast Asia (Haddow et al., 2012). However, in 2007 for the first time ZIKV was reported outside of Africa and Southeast Asia in Yap Island (Hayes, 2009). In Yap Island out of the 185 suspected cases 49 of them were confirmed to be ZIKV and majority of the cases occurred in the older age group (50–55 years) (Duffy et al., 2009). However, during that outbreak there was no death or haemorrhagic complications and the patients only suffered from symptoms like rash, fever, arthritis or arthralgia, conjunctivitis, myalgia, headache, retro-orbital pain, edema, and vomiting (Duffy et al., 2009).

In recent years 2012–2014 there were outbreaks of ZIKV in the Pacific Islands namely Cook Island, Easter Island, French Polynesia, and New Caledonia (Cao-Lormeau and Musso, 2014; Roth et al., 2014). In some of the Pacific Islands especially in 2014 all three viruses ZIKV, chikungunya virus (CHIKV), and dengue virus (DENV) were circulating (Cao-Lormeau and Musso, 2014). These viruses showed a gradual spread over the years starting in 2007 and becoming more widespread in 2014. It is of great concern to learn that in French Polynesia when 1505 asymptomatic blood donors were screened for ZIKV by RT-PCR, 42 of them were positive (Musso et al., 2014). This seems to implicate how travel by humans can help to spread the viruses to new areas.

From February to April 2015, north eastern states of Brazil reported almost 7000 cases of people having rash and minor illness; of which only a small percentage of them were positive for dengue while tests for other viruses (but not for ZIKV) were all negative (Kindhauser et al., 2016). It was only by early May 2015 it was confirmed that it was ZIKV by RT-PCR and was reported for the first time in the Americas (Kindhauser et al., 2016). By July 12 states in Brazil had confirmed ZIKV cases and by the end of 2015 Colombia, Suriname, El Salvador, Mexico, Guatemala, Paraguay, Venezuela, Honduras, and Panama had reported locally acquired ZIKV (Kindhauser et al., 2016). This implies that the ZIKV will go on spreading to many more countries unless concerted effort is taken on a global scale.

Zika virus which was thought to be just a mild viral disease was later found to cause neurologic symptoms and microcephaly (Oliveira Melo et al., 2016). ZIKV was also found in other body fluids and was also shown to be sexually transmitted (Musso et al., 2015; Mansuy et al., 2016; Venturi et al., 2016). The current situation seems to portray that ZIKV could lead to serious public health concerns on a global scale. In the Americas ZIKV has been circulating along with DENV and CHIKV.

In Southeast Asia it is known that arbovirus diseases like DENV, CHIKV, Japanese encephalitis are serious public health concerns (Dash et al., 2013). In recent years (2014–2015) in Indonesia, a positive case of ZIKV was detected during a dengue outbreak in Jambi province Sumatra (Perkasa et al., 2016). Similarly in Cambodia a confirmed case of ZIKV was reported in 2010 (Heang et al., 2012) and in 2012 in Cebu, Philippines a 15 year old boy was confirmed to be suffering from ZIKV by real time RT-PCR and virus isolation (Alera et al., 2015). Travelers to Thailand were found to be infected with ZIKV on return to their country (Tappe et al., 2014). The Thai Ministry of Health then reviewed cases and found ZIKV infection circulating in Thailand between 2012 and 2014 (Buathong et al., 2015). Due to large outbreaks of dengue and CHIKV in Southeast Asia, which cause similar symptoms, ZIKV may be overlooked in Malaysia (Sam et al., 2016). However, there have been no reports of other neurologic symptoms.

This review will delve into the methods available for the detection of ZIKV, the vectors involved, current tools used for the control of the vectors and finally on the recommendations of new paradigms for surveillance and control of these vectors.

## Diagnostic Tools of ZIKV

Dengue virus and CHIKV share the same mosquito vectors (*A. aegypti* and *A. albopictus*) and potential distribution as ZIKV, and indeed co-circulation is described in the Americas (Rodriguez-Morales et al., 2016). It is difficult to clinically differentiate between these infections as there is much overlap in symptoms and signs. Laboratory diagnosis takes on added importance as the long-term consequences of these infections are quite different and require specific approaches, for example the follow-up of ZIKV-infected pregnant women. There has been a flurry of new diagnostic assays described recently to complement existing conventional techniques such as cell culture [reviewed by Waggoner and Pinsky (2016)]. To date (July 20, 2016), several PCR and IgM assays for ZIKV have been submitted to the WHO Emergency Use Assessment and Listing Procedure, which assesses and expedites the availability of *in vitro* diagnostics during public health emergencies (World Health Organization, 2016a).

The current gold standard for diagnosis is PCR, which should be carried out on serum samples (within 7 days of illness) or urine (within 14 days) (CDCP, 2016). ZIKV RNA can also be detected in saliva (Bingham, 2016) and semen (Reusken et al., 2016) (the latter for up to 62 days), and there is some evidence that the non-serum specimens urine, saliva, and semen may be more likely to yield positive results than serum (Bingham, 2016; Reusken et al., 2016). Serum samples should also be tested for co-circulating arboviruses such as DENV and CHIKV (Waggoner et al., 2016).

Detection of serum IgM from day five of illness onward is a mainstay for arboviral diagnosis in most diagnostic laboratories in developing countries, as culture and PCR facilities are not widely available. ZIKV IgM can also be detected in the CSF of babies with microcephaly suspected to be due to congenital ZIKV infection (Cordeiro et al., 2016). However, the utility of IgM is much reduced by the extensive cross-reactions seen with past infections of or vaccinations against other flaviviruses, notably DENV, Japanese encephalitis, and yellow fever viruses (Calisher et al., 1989), necessitating the use of the highly specific plaque reduction neutralization test for confirmation (Lindsey et al., 1976; Rabe, 2016). This assay is beyond the scope of most laboratories. The antibodies that cross-react to ZIKV or DENV are mainly targeted to envelope protein domains EDI/II, and can cause antibody-dependent enhancement of infection with either virus (Stettler et al., 2016). In contrast, antibodies to non-structural protein 1 (NS1) are ZIKV-specific and could be used to develop a serological assay that can distinguish DENV from ZIKV infections (Huzly et al., 2016; Stettler et al., 2016). However, negative test results by culture, PCR or serology can never fully rule out ZIKV infection.

The ideal diagnostic test for ZIKV should be affordable, sensitive, specific, user-friendly, rapid and robust, particularly for the developing countries where the vectors exist. One of the WHO’s top priorities for ZIKV medical products are multiplex tests for the three arboviruses (ZIKV, DENV, and CHIKV) which share the same mosquito vectors (World Health Organization, 2016b). The ideal test should detect RNA or antigen. The development of NS1 antigen detection assays (including rapid tests) was a major advance for dengue diagnosis. NS1 is secreted by flavivirus-infected cells and is involved in immune evasion and pathogenesis. ZIKV NS1 shares conserved features with DENV and West Nile virus, but has different electrostatic potential at the loop surface, which interacts with host factors and antibodies (Song H.et al., 2016). Unlike IgM assays, DENV NS1 assays do not seem to demonstrate cross-reactivity with ZIKV (Matheus et al., 2016), apart from a single case report using a particular kit (Gyurech et al., 2016). A ZIKV NS1 assay would theoretically be feasible as an accessible test to reliably differentiate DENV and ZIKV, and several candidate assays are in the pipeline (World Health Organization, 2016b).

Several alternative diagnostic field tools for resource-poor settings have been described (Meagher et al., 2016). These include a synthetic biology approach, whereby isothermal RNA amplification is carried out, and toehold switch RNA sensors induce a color change, with all reagents embedded into a paper-based sensor (Pardee et al., 2016). A point-of-care loop-mediated, isothermal amplification assay with colorimetric detection has also been described (Song J.et al., 2016).

The detection of arboviruses in wild mosquitoes is useful for surveillance or for identifying the vectors of a relatively understudied pathogen (such as ZIKV) (Samuel and Tyagi, 2006). However, there are specific challenges which reduce sensitivity of testing methods. For example, mosquitoes may not be collected from traps for some time, which will lead to drying, rapid loss of viability for culture, and RNA degradation. Pools of triturated mosquitoes may also contain PCR inhibitors and other microorganisms, which may contaminate cultures. The traditional culture techniques for arbovirus diagnosis in mosquitoes, such as inoculation in cells, suckling mice or mosquitoes, and immunofluorescence assay, are in any case too labor-intensive for routine surveillance. Next-generation sequencing is useful for mosquitoes which potentially carry more than one pathogen or during an outbreak with an unknown arbovirus, but it is expensive and requires complex bioinformatics analysis (Bishop-Lilly et al., 2010).

For DENV, the rapid commercial NS1 assays developed for human diagnosis are excellent tools for testing mosquitoes, with the benefits of similar sensitivity to PCR (Tan et al., 2011; Voge et al., 2013), simplicity, and the potential for field use with a hand-held battery-operated homogenizer (Muller et al., 2012). Antigen-capture enzyme immunoassays have been described for detection of other flaviviruses in desiccated mosquitoes kept at ambient temperatures, including DENV (Thenmozhi et al., 2005; Chao et al., 2015) and Japanese encephalitis virus (Tewari et al., 1999). The surveillance of mosquitoes is a potential additional application for future ZIKV antigen assays.

## Vectors of ZIKV

*Aedes aegypti* and *A. albopictus* are known to be the vectors of ZIKV (Li et al., 2012; Wong et al., 2013) and these two mosquitoes are also responsible for transmission of DENV and CHIKV. These are container breeding mosquitoes and it is known that the eggs of these mosquitoes can withstand desiccation. Thus *Aedes* mosquitoes are easily dispersed to many areas. It is also known that *A*. *aegypti* exhibits skip oviposition where it deposit its eggs in many containers (Reiter, 2007).

Zika virus was first isolated from *A. aegypti* from the rural area of Bentong in Pahang, Malaysia in 1965 (Marchette et al., 1969). Recent studies carried out in Singapore demonstrated that *A. aegypti* was susceptible to ZIKV and by day five almost 60% of the mosquito’s salivary glands were positive and on day six 100% were positive (Li et al., 2012). Studies conducted by the same group also demonstrated that *A*. *albopictus* could transmit ZIKV and by day 10 100% transmission was obtained in mosquito’s saliva (Wong et al., 2013).

Zika virus was found naturally infected in *A. aegypti* in 1965 (Marchette et al., 1969) and seropositivity of ZKIV was also reported in 1960s (Dash et al., 2013). Thus, is it possible that the ZIKV has been in Southeast Asia all the time and people have developed immunity to this virus? It has been postulated that ZIKV originated in East Africa and spread to West Africa and Asia thus forming three different genotypes; the Asian genotype further spread to Pacific Islands and the Americas (Lanciotti et al., 2016). Also a case of ZIKV was confirmed in a traveler who visited Sabah, Malaysian Borneo on his return to Germany (Tappe et al., 2015). Thus, there must be other cases that have not been reported, perhaps people would only have suffered mild symptoms and it would not have been detected.

In Gabon there was an outbreak of CHIKV and DENV in 2007 and 2010 (Grard et al., 2014). The predominant vector found was *A. albopictus* and 91 pools of them were screened of which four pools were positive for CHIKV, three pools for DENV and two pools had mixed infection of CHIKV and ZIKV (Grard et al., 2014). When sera samples from humans were screened five were found to be positive for ZIKV (Grard et al., 2014). Here it clearly showed that ZIKV was circulating along with CHIKV and DENV. By screening both human and mosquito pools concrete evidence has been established that ZIKV can be transmitted alongside CHIKV and DENV. This clearly indicates that the trapping of adult mosquitoes and detection of viruses in them is the way forward to prevent epidemics.

It has been estimated that 440,000 to 1,300,000 ZIKV cases have occurred in Brazil (Bogoch et al., 2016), and the virus has finally been isolated from *A. aegypti* in that country (Gretchen, 2016). Now that *A. aegypti* can be easily trapped using the sticky gravid trap, this should be carried out and the vector should be confirmed in all localities. With such a large number of cases one would expect that it would be fairly easy to obtain infected mosquitoes. For example in a dengue prone area in Selangor, Malaysia, we obtained a minimum field infection rate (MIR) of 38.02 per 1000 using the NS1 antigen test kit (Lau et al., 2015). Since it is more difficult to get blood from people living in urban areas and it involves ethical clearance the best way to move forward is to detect the virus in the mosquitoes and to start proper control measures when results are positive.

The same vectors *A. aegypti* and *A. albopictus* are responsible for the spread of DENV and CHIKV and these vectors know no borders. If control measures can be instituted for these vectors the incidence of all these arboviral diseases will also be decreased. It seems like ZIKV is taking the same route as CHIKV (Musso and Gubler, 2015). If that is the case Southeast Asia could be in the forefront for ZIKV outbreak in the very near future. Perhaps the people of Southeast Asia are already immune to the disease, but visitors to the region may get infected and help to spread the disease globally.

## Vector Control Measures

Vector control measures carried out in Southeast Asia for surveillance and control of *A. aegypti* are shown in **Table 1**. It can be seen that vector surveillance and control strategies are mainly targeting the larval breeding sites. This includes the use of chemicals (Lee et al., 1997; Sulaiman et al., 2000, 2002; Chung et al., 2001; Tun-Lin et al., 2009; Huy et al., 2010; Oo et al., 2011; Kittayapong et al., 2012; Saiful et al., 2012; Sommerfeld and Kroeger, 2012), biological agents (Seng et al., 2008a; Lacroix et al., 2012; Hugo et al., 2014; Lazaro et al., 2015; Zuharah et al., 2015), environmental management (Ooi et al., 2006; Tun-Lin et al., 2009; Lee et al., 2013; Lau et al., 2015), and community participation (Chang et al., 2011). We have become over-dependent on chemicals and now the *Aedes* mosquitoes are resistant to most pyrethroids (Ponlawat et al., 2005; Wan-Norafikah et al., 2010; Koou et al., 2014a,b; Ishak et al., 2015). Studies have also shown that space spraying has not conclusively been effective in reducing dengue transmission (Mount, 1998; Perich et al., 2000; Esu et al., 2010). Thus, when fogging/ULV is carried out impact on the vectors is minimal as shown in some studies (Vythilingam and Panart, 1991; Tanrang and Vythilingam, 2004). This could be one reason why cases of DENV are on the increase in countries in Southeast Asia.

**Table 1:** Current research on *Aedes* vector control in Southeast Asian over the past 25 years.

Countries	Environmental	Chemical	Biological
Cambodia		Temephos (Huy et al., 2010). Pyriproxyfen (Seng et al., 2008b)	Guppy larvivorous fish (Seng et al., 2008a)
Indonesia		Transfluthrin/metofluthrin (Achee et al., 2015)	Mosquitoes with *Wolbachia* (Rašić et al., 2015)
Laos			*Mesocyclops* spp. (Lazaro et al., 2015)
Malaysia	Sticky traps (Lau et al., 2015)	*Bacillus thuringiensis israelensis* (Saiful et al., 2012). Pyriproxyfen (Vythilingam et al., 2005). Cynoff 25ULV (cypermethrin 25 g/l) and Solfac UL015 (cyfluthrin 1.5% w/v) (Sulaiman et al., 2002). Deltamethrin/S-bioallethrin/piperonyl butoxide and cyfluthrin (Sulaiman et al., 2000). ULV-applied bifenthrin (Lee et al., 1997)	Mosquitoes carrying a Dominant Lethal gene, RIDL (Lacroix et al., 2012). *Toxorhynchites splendens* (Zuharah et al., 2015)
Myanmar		*Bacillus thuringiensis* subsp. *Israelensis (*Oo et al., 2011). Temephos (Tun-Lin et al., 2009)	Dragonfly nymphs and fish (Tun-Lin et al., 2009)
Philippines		Pyriproxyfen (Aumentado et al., 2015). *Bacillus thuringiensis* subsp. *Israelensis* (Sommerfeld and Kroeger, 2012)	Guppy (Chang et al., 2011)
Singapore	Mosquito traps (Lee et al., 2013)	*Bacillus thuringiensis* and chemical fogging (Chung et al., 2001)	
Thailand	Screen net covers, mosquito traps, vacuum aspirators (Kittayapong et al., 2012) and treated curtains (Lenhart et al., 2013); Insecticide treated clothing (Tozan et al., 2014). Mosquito trap (Ponlawat et al., 2013)	Temephos (Kittayapong et al., 2012). *Bacillus thuringiensis* subsp. *Israelensis* (Kittayapong et al., 2012).	Copepods (Kittayapong et al., 2012)
Vietnam	Olyset Nets (Tsunoda et al., 2013)	Pyriproxyfen (Tsunoda et al., 2013)	*Mesocyclops* sp (Lazaro et al., 2015). Mosquito with *Wolbachia* (Nguyen et al., 2015)

Chang et al. (2011) have suggested that a positive move would be to include all three parameters of dengue transmission – vector density, human cases, and vector infection rate for prediction of early outbreaks. This seems to be the way forward in controlling these arboviral diseases. It has also been demonstrated that the gravid *Aedes* mosquito can be easily captured using sticky traps and the infected mosquito was obtained before human cases were reported (Lau et al., 2015). Studies conducted in different countries have demonstrated that the sticky traps are effective in collecting the *Aedes* mosquitoes when they come to oviposit (Ritchie et al., 2004; Gama et al., 2007; Honório et al., 2009; Chadee and Ritchie, 2010; de Santos et al., 2012; Resende et al., 2013). Thus a proactive approach is needed to test these mosquitoes for the different viruses so that a more positive control approach can be instituted. In Singapore too it has been shown that the sticky trap was able to trap the infected *Aedes* mosquito (Lee et al., 2013). It has also been documented that asymptomatic cases were infectious to *Aedes* mosquitoes and thus silent transmission was ongoing all the time (Duong et al., 2015).

House to house larval surveys have been the hallmark of dengue control program in many countries in Southeast Asia (Cheong, 1967; Ho and Vythilingam, 1980; Cheong et al., 1986; Chang et al., 2011; Mudin, 2015; Hapuarachchi et al., 2016). This method has also been used for the control of CHIKV vectors and can be used for ZIKV vectors. However, current studies have shown that although the *Aedes* house index has been reduced to as low as 0.07–0.14, yet epidemics of dengue have been explosive as reported in Singapore (Hapuarachchi et al., 2016). This has been due to the switch in serotypes as noted in 2007–2008 outbreaks (Lee et al., 2010) and also in 2013–2014 (Hapuarachchi et al., 2016). Singapore being a small and an aﬄuent country can afford to carry out serotyping and sequencing in a timely manner but still face epidemics of dengue. It is agreed that a multi-pronged approach backed by the epidemiological, virological, and entomological understanding is necessary for the control of vector borne viral diseases. However, entomological activities have always been reactive and thus could be one of the reasons for the current epidemics in many countries.

## Biological Control

The biological control approach traditionally reduce vector numbers by means of introducing their natural predators, such as larvivorous fish (Seng et al., 2008a) dragonfly nymphs (Tun-Lin et al., 2009), *Mesocyclops* sp (Lazaro et al., 2015), and *Toxorhynchites splendens* (Zuharah et al., 2015). While these approaches are environmentally friendly, they only affect the immature stages of the mosquito vector. In addition, they are effective only in containers that are constantly filled, such as wells and large containers (World Health Organization, 2012). Now that most breeding sites are cryptic it will be difficult to use biological control.

## Insect Growth Regulator

Pyriproxyfen an insect growth regulator has been tested under field conditions and it has a unique mode of action where it inhibits the development of the adult mosquito and also when an adult mosquito comes in contact with pyriproxyfen it can help to transfer it to other containers (Invest and Lucas, 2008). Studies carried out in Southeast Asia have shown that low doses were required for the inhibition of adult *Aedes* and the residual activity can be maintained for 11–15 weeks (Vythilingam et al., 2005) in Malaysia, while in Cambodia using a slow release formulation residual activity was effective for 6 months (Seng et al., 2008b). In Philippines pyriproxyfen was successfully used to control the dengue outbreak after typhoon Haiyan (Aumentado et al., 2015).

## The Way Forward

If we learn lessons from malaria control with regards to vectors, it was always targeted toward adult mosquitoes and not so much against the larvae. One reason could be that because it was difficult to find the breeding sites of *Anopheles* mosquitoes and some sites were inaccessible. However, for dengue, the vectors breed in containers and thus control of larvae and source reduction were the initial strategies for dengue control. This has obviously worked since *Aedes* index has been reduced to low levels (Mudin, 2015; Hapuarachchi et al., 2016) yet the cases of DENV infection have increased. Thus, it is timely now to focus on interventions based on the adult population to reduce and prevent epidemics of DENV. If we control the *Aedes* adults, we will automatically reduce outbreaks of ZIKV and CHIKV.

Besides monitoring the adult *Aedes* population it is similarly important to detect the pathogen in the mosquitoes. Currently for dengue the NS1 antigen test kit can be used and the procedure is very simple for use by public health workers. Thus what is needed for CHIKV, ZIKV, and other common arthropod borne viruses are very simple tools that can be used by the public health workers. Molecular tools like PCR and real-time PCR are available but these are expensive and need experienced staff and expensive equipment which is not feasible for a control program.

Although it has been stated that in Americas the success of *A. aegypti* eradication was due to perifocal spraying and source reduction (Achee et al., 2015), this will not work in present times because currently it is difficult to persuade people in urban areas to allow indoor residual spraying to be carried out. Besides in Southeast Asia it is also known that the *A. aegypti* like to rest on temporary surfaces like clothes and curtains (Pant and Yasuno, 1973).

Field studies have been carried out on use of insecticide treated curtains and jar covers and these have shown reduction in mosquito population (Kroeger et al., 2006; Lenhart et al., 2013; Tsunoda et al., 2013), however, its efficacy in reducing dengue cases have not been deciphered. Thus although a number of studies were conducted on various methods to monitor adult population (Lee et al., 2013; Ponlawat et al., 2013; Tozan et al., 2014; Lau et al., 2015) and show promise, the end result of reduction of cases has not been established.

Studies are also on going on genetically modified mosquitoes and one showing promise is the release of insects with dominant lethality (RIDL). In the laboratory these mosquito larvae are bred in water containing tetracycline, however, in the absence of tetracycline these larvae and pupae will not be able to survive. Field studies have been conducted in Cayman Islands, where there was a suppression of 80% of the natural population (Harris et al., 2012) and in Brazil there was a suppression of 85% of the natural population (Achee et al., 2015). Although this method has reduced mosquito populations, it should be noted that only male mosquitoes were released. However, a fool proof method is needed to ensure that females are not released. RIDL females are equally susceptible to dengue virus compared to the wild *A. aegypti*. Besides, evidence is required to show that with the reduction of the *A. aegypti* population it is possible to reduce dengue cases. Each country will also need to obtain approval from regulatory bodies before they can release these mosquitoes. Insectaries will also have to be maintained if these RIDL mosquitoes are to be used. All these come with a cost and countries should be able to afford these expensive methods before they embark on such a program.

Another similar approach is the release of *A. aegypti* with the bacteria of *Wolbachia* sp. *Wolbachia* is naturally found in many arthropods and nematodes but is not found in *A. aegypti* (Werren, 1997). Currently, one of the novel approaches for bio-control is the introduction of *Wolbachia* from naturally infected arthropods into *A. aegypti* to reduce dengue transmission (Moreira et al., 2009; Walker et al., 2011). When an uninfected female *A. aegypti* mates with a *Wolbachia-*infected male, the female will produce eggs but no progeny will develop due to cytoplasmic incompatibility. However, when a *Wolbachia* infected female mates with either infected or uninfected male, all progeny will carry *Wolbachia* (Caragata et al., 2016). Field trials have been conducted in Australia with the release of *A. aegypti* with wMel *Wolbachia* and the frequency has remained at more than 90% for 3 years (Hoffmann et al., 2014). However, field release of *A. aegypti* with wMelPop *Wolbachia* in Vietnam and Australia failed to become successfully established (Nguyen et al., 2015). Studies are also ongoing in Indonesia (Rašić et al., 2015). Thus it will take time for more studies to be conducted before *Wolbachia* infected *A. aegypti* can be used in dengue control program.

Now with ZIKV becoming a huge public health global problem, it is timely that randomized control trials (RCT) need to be carried out in Southeast Asia and prove that some of these paradigms will be able to control and prevent epidemics caused by these *Aedes* mosquitoes. For a start RCT studies should show that the cases of dengue can be reduced (Reiner et al., 2016). If a particular paradigm proves to be successful, it would also work for all the other arboviruses transmitted by *A. aegypti* and *A. albopictus*.

## Conclusion

There are several options for ZIKV diagnosis building on existing technologies, which can be used in both humans and mosquitoes. However, most are not available in developing countries, and there remains an urgent need for an accessible RNA/antigen assay, as well as an IgM assay with acceptable specificity against other flaviviruses. While extensive work is ongoing to develop a vaccine, diagnostic kits, and to study the epidemiology of ZIKV, it is equally important to develop new paradigms to control the vectors. We need to learn from the past and thus a more proactive approach is needed to control the vectors and not a reactive one. In the early years besides Africa, ZIKV was known to be circulating Southeast Asia. Thus it is imperative to ensure that Southeast Asia don’t become a hub for transmitting the ZIKV to other countries. We need to work together and carry out multi-country RCT for vector control to show that the way forward is to monitor the adult *Aedes* population along with infectious status.

## Author Contributions

IV, JS, YC, LK, and WW all played a role in the preparation of this manuscript.

## Conflict of Interest Statement

The authors declare that the research was conducted in the absence of any commercial or financial relationships that could be construed as a potential conflict of interest.
